# Topological data analysis in medical imaging: current state of the art

**DOI:** 10.1186/s13244-023-01413-w

**Published:** 2023-04-01

**Authors:** Yashbir Singh, Colleen M. Farrelly, Quincy A. Hathaway, Tim Leiner, Jaidip Jagtap, Gunnar E. Carlsson, Bradley J. Erickson

**Affiliations:** 1 Mayo Clinic, Rochester, MN USA; 2 Staticlysm LLC, Miami, FL USA; 3 School of Medicine, West Virginia University, Morgantown, WV USA; 4 Department of Mathematics, Stanford University, Stanford, CA USA

**Keywords:** Topological data analysis, Persistent homology, Medical imaging, Texture landmarks

## Abstract

Machine learning, and especially deep learning, is rapidly gaining acceptance and clinical usage in a wide range of image analysis applications and is regarded as providing high performance in detecting anatomical structures and identification and classification of patterns of disease in medical images. However, there are many roadblocks to the widespread implementation of machine learning in clinical image analysis, including differences in data capture leading to different measurements, high dimensionality of imaging and other medical data, and the black-box nature of machine learning, with a lack of insight into relevant features. Techniques such as radiomics have been used in traditional machine learning approaches to model the mathematical relationships between adjacent pixels in an image and provide an explainable framework for clinicians and researchers. Newer paradigms, such as topological data analysis (TDA), have recently been adopted to design and develop innovative image analysis schemes that go beyond the abilities of pixel-to-pixel comparisons. TDA can automatically construct filtrations of topological shapes of image texture through a technique known as persistent homology (PH); these features can then be fed into machine learning models that provide explainable outputs and can distinguish different image classes in a computationally more efficient way, when compared to other currently used methods. The aim of this review is to introduce PH and its variants and to review TDA’s recent successes in medical imaging studies.

## Key points

1. Topological data analysis (TDA) provides information on the shape of data.
2. In radiology, the shape of 2D and 3D images contains additional information.
3. TDA can be combined with other applications, such as textural analysis.
4. Persistent homology can provide a visual representation of extracted TDA data.

## Background

Over the last few years, rapid advancements in artificial intelligence and deep learning, in particular, have resulted in a surge of publications in medical image analysis fields. Establishing innovative, effective diagnostic support tools could improve disease detection, such that physicians can make more accurate diagnostic decisions to quickly treat patients [1, 2]. Physical exam findings, laboratory testing, and expert-driven interpretation of ultrasonography, computed tomography (CT), and magnetic resonance imaging (MRI) are used in clinical practice for detecting a variety of conditions. Medical imaging datasets are now commonplace in the biomedical industry. However, healthcare image data collection such as CT or MRI datasets may involve high dimensionality (many predictors relative to patient samples) or mismatches in metric scale from equipment calibration differences, both of which can pose issues for deep learning and other machine learning algorithms [3, 4].

Topological data analysis (TDA) is a novel approach to medical imaging analytics that leverages tools from topology, a branch of mathematics that can look at global structures in data, such as loops or holes, that do not depend on specific measurements, such that features exist irrespective of whether they are measured in centimeters, inches, or other units. TDA solves the issues of dimensionality (the large number of predictors relative to the number of patients from whom data was collected) and metric mismatches (such as the aforementioned unit of measurement). In a coordinate-free approach (where metrics are not needed or used), this branch of data science defines the dataset structure as shapes; these shapes are created by connecting pieces of point data or loops within the dataset, profiling the data as point clouds with a notion of distance or similarity [1–4]. Datasets collected on the same biological systems using different technological platforms can thus be directly compared. In addition, TDA is well suited to deal with the high dimensionality present in medical imaging and biological analyses [5]. TDA has been studied in a variety of medical fields including neurology, cardiology, hepatology, gene-level and single-cell transcriptomics, drug discovery, evolution, and protein structural analysis [6]. TDA has been successfully utilized in a variety of medical contexts, such as the identification of novel pathological phenotypes of asthma, the discovery of phenotype-biomarker associations in traumatic brain injury [5], the identification of diagnostic factors for pulmonary embolism [7], and the differentiation between healthy patients and those with diabetic retinopathy from retinal imaging [8].

TDA has been combined with convolutional neural networks (CNNs) to improve the analysis of radiomics data. In fact, CNNs themselves are a type of topology-based algorithm that optimizes mapping between topological spaces with respect to an outcome and input data [9, 10]. However, training CNNs still requires large samples of imaging data and enough images within each outcome group of interest to allow the CNN to find relevant feature differences that distinguish the groups. TDA can work with very small sample sizes and find meaningful information, allowing for its use in cases where CNNs may not have enough data to create an accurate model. In fact, CNNs themselves are a type of topology-based algorithm that optimizes mapping between topological spaces with respect to an outcome and input data.

Given the continued expansion of data acquisition due to the development of next-generation high-throughput sequencing [11], high dimensional medical imaging such as spectral CT and MRI [12–14], and a greater emphasis on personalized medicine [15, 16], effective data analysis methods are essential for transforming this data into information that may be used in clinical diagnostic and therapeutic settings. The goal of TDA is to identify regional and global structures in data at various scales by concentrating on the shape of the data to solve the issues of data dimensionality and differences in data collection methodologies and scales.

## Brief overview of persistent homology

Persistent homology (PH) is a commonly used tool from TDA that relies on two notions: (1) filtration with a distance metric (so that we can create a series of data objects from the initial object) and (2) tracking topological features over that filtration (so that we can examine the shapes that exist in each of the data objects created by the filtration). After providing a detailed framework of the statistical basis of this approach, we provide a practical example to help illustrate the properties of PH.

To filter a data point cloud or distance matrix built from a point cloud, a series of threshold distances is defined with a metric, such that each threshold iteratively cuts the dataset and builds a topological object from that defined threshold distance. Essentially, points that are within the thresholded distance of each other (either pairwise in the Vietoris–Rips complex or all mutually in the Cech complex) are connected into an object (called a simplicial complex) with vertices and edges and higher-dimensional analogues of edges (such as faces). A graph is a simple example of a simplicial complex with mutual two-way connections between points. However, if three points are mutually within a distance threshold of each other, they are connected into a triangle. If four points are mutually within a distance threshold of each other, they form a tetrahedron. This pattern holds to an arbitrary number of points that are within a mutual distance of each other. As this process is applied to each threshold, a series of simplicial complexes is created with a hierarchical structure.

**Fig. 1:**
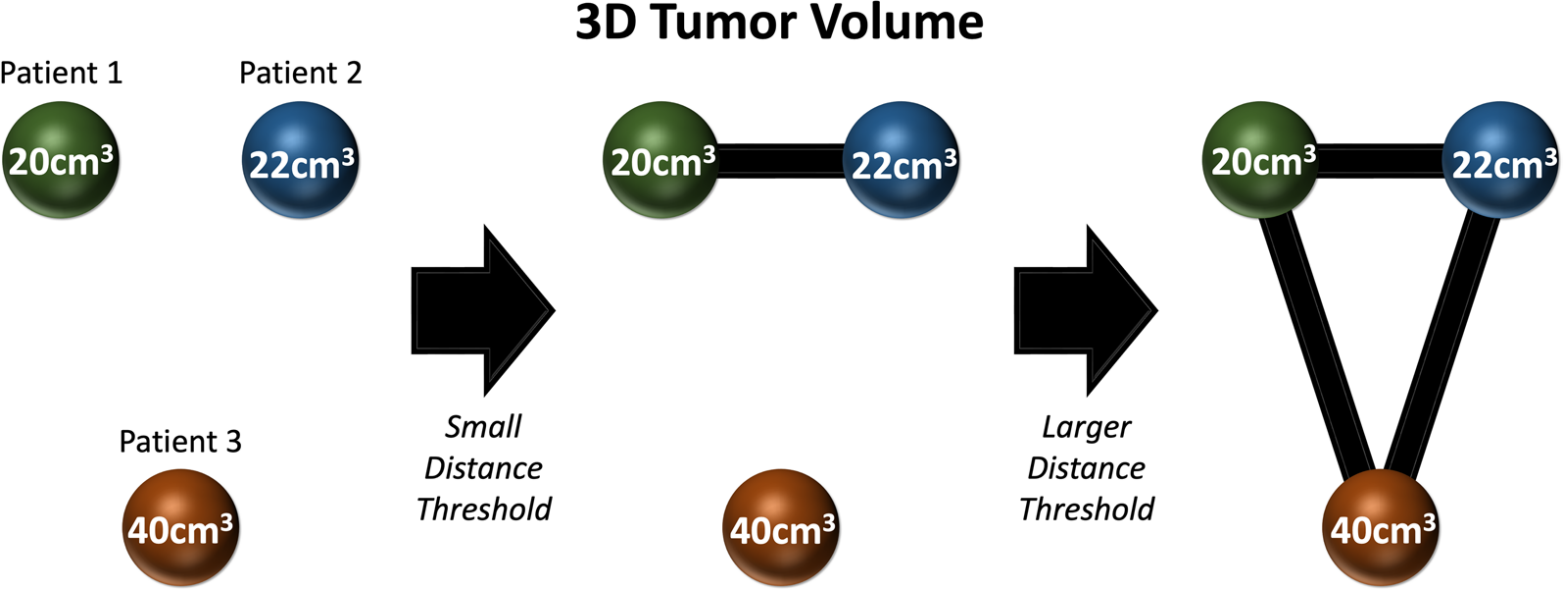
An example of three patients with varying 3D tumor volumes within a two-dimensional point cloud. The point from patient 1 and patient 2 for 3D tumor volume is close, therefore only requiring a small distance threshold to create a simplicial complex. As the distance threshold is expanded, the simplicial complex can include additional points with increasing variance

For example, suppose we have three points in a two-dimensional point cloud, two of which are closer together than either is to the third point (Fig. 1). In this example, we can take three patients who present with malignancy, each having a different 3D tumor volume on CT imaging. For patients 1 and 2, their volumes are similar and form a simplicial complex with only a small distance threshold. If we expand our distance threshold, we can now include other values that are increasing dissimilar, connecting all three points to create a triangle. In practice, we’d have many more points and dimensions in our point cloud, but this illustrates the principle of filtration and simplicial complex hierarchies at a basic level.

Once the series of simplicial complexes has been built, their structures can be analyzed. Homology, a topological tool, counts the number of holes in each dimension that exist within a space. In simplicial complexes, these are connected components, loops, voids, and higher-dimensional voids. To build the intuition around this, it is important to consider a part in a simplicial complex made of three, two-way relationships (edges in a graph) but lacking a mutual three-way relationship (to form a triangle of three points within a mutual distance of each other). This forms a loop, a part of the space with a potentially higher-dimensional interaction that does not exist in the simplicial complex but includes the lower-dimensional interactions. We can consider mutual three-way relationships that do not form a mutual four-way relationship, which would create a void. Betti numbers, which track the number of holes that exist in each dimension for a space or a simplicial complex, are a good way to summarize and quantify this information.

**Fig. 2:**
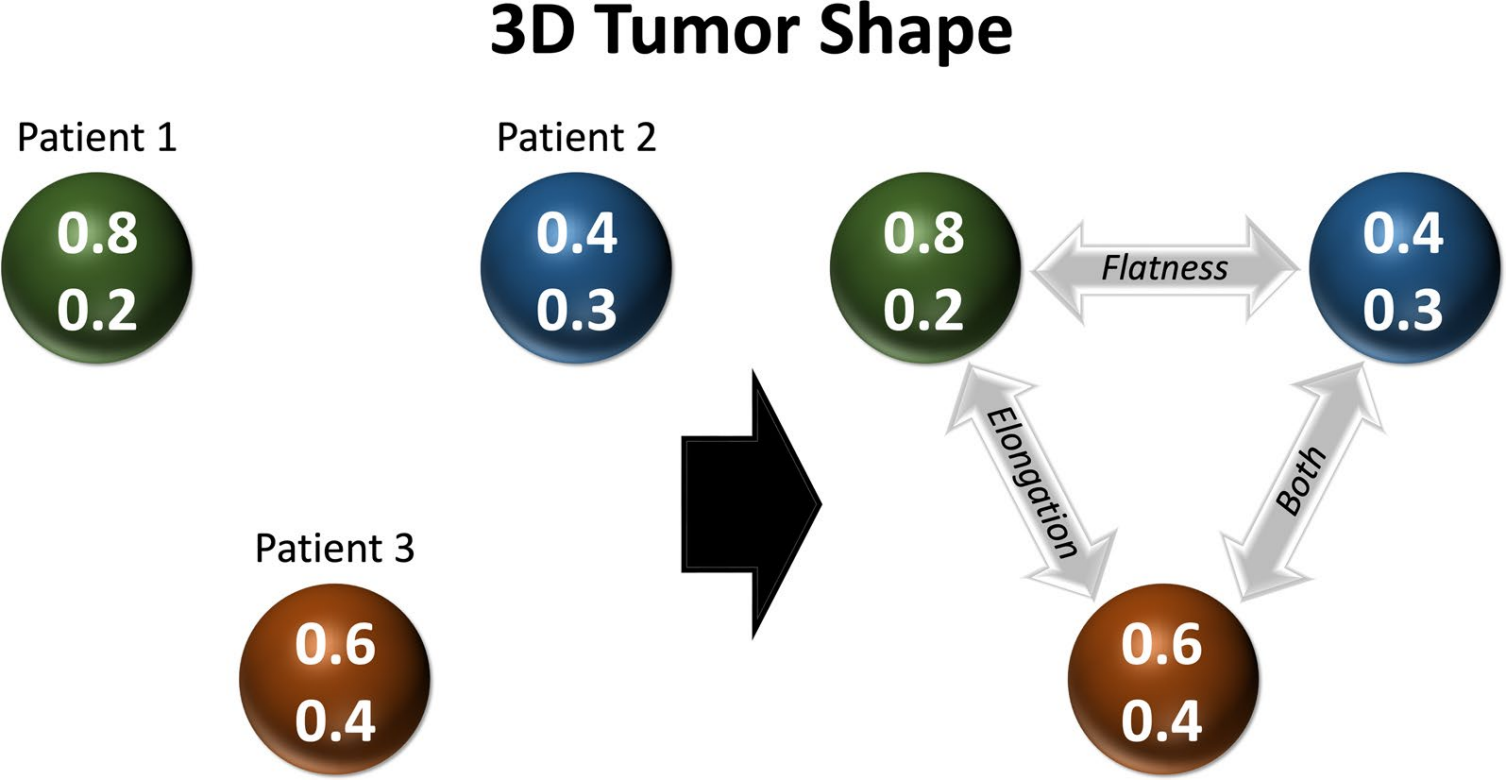
An example of three patients with varying 3D tumor shapes within a two-dimensional point cloud. In examining characteristics such as elongation and flatness, the points form mutual two-way relationships within a distance or filter characteristic without a three-way relationship. This can be altered by the data scientist, as increasing the distance threshold can increase the points included within a simplicial complex

In Fig. 2, we have three two-way relationships that mutually exist (perhaps distance or some other metric upon which we’ve filtered the data); however, the criterion for a three-way relationship does not exist, though all three, two-way relationships do exist. In this example, we use characteristics comprising the shape of a tumor identified from CT imaging, such as elongation and flatness, to demonstrate the principle of three two-way relationships that can mutually exist. While each of the pairs share aspects of elongation, flatness, or both qualities, the criterion for creating a three-way relationship is not met; this creates a loop where a three-way relationship currently does not exist but has the potential of existing under varying conditions assigned by the data scientist.

**Fig. 3:**
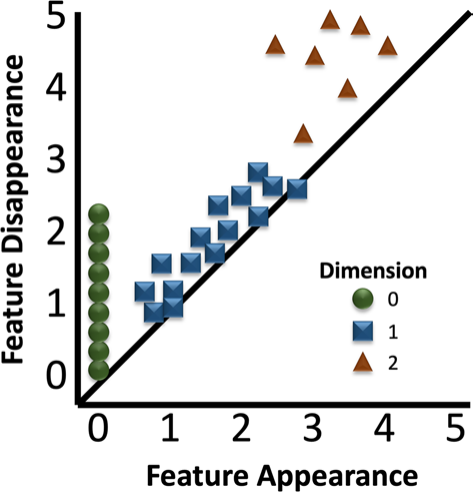
Persistence diagram that tracks features in the 0th, 1st, and 2nd homology groups. This persistence diagram shows where the 0th, 1st, and 2nd Betti numbers appear (*X*-axis) and disappear (*Y*-axis) throughout the filtration of the data

Because we have a series of simplicial complexes from our filtration, we can track the Betti numbers for holes in each dimension across the filtration to see where topological features appear and disappear in the dataset. This provides us with information about which features might be most important (features that persist across a large part of the filtration) and those which might just be noise in the dataset (features that do not persist long across the filtration). These features and their lifetimes within the filtration can be plotted visually with a persistence diagram. This allows for the identification of features in medical images and potential comparison of features across different datasets. In fact, there are distance metrics that can measure this difference in features directly; Wasserstein distance, also known as earth mover distance, is the most applied metric. Figure 3 is a simple example of a persistence diagram that tracks features in the 0th, 1st, and 2nd homology group (connected components, the 0th, 1st, and 2nd Betti numbers).

Compute times for persistent homology are reasonable for medical images. On a basic laptop, one fMRI image takes about 1–2 min to process with the TDApplied [17]. With a distributed computing system of 20 cores, 5000 fMRI images would take about 250–500 min (~ 4–8 h) to process. On a GPU system with hundreds of cores, it is possible to scale this estimate across large healthcare systems.

**Fig. 4:**
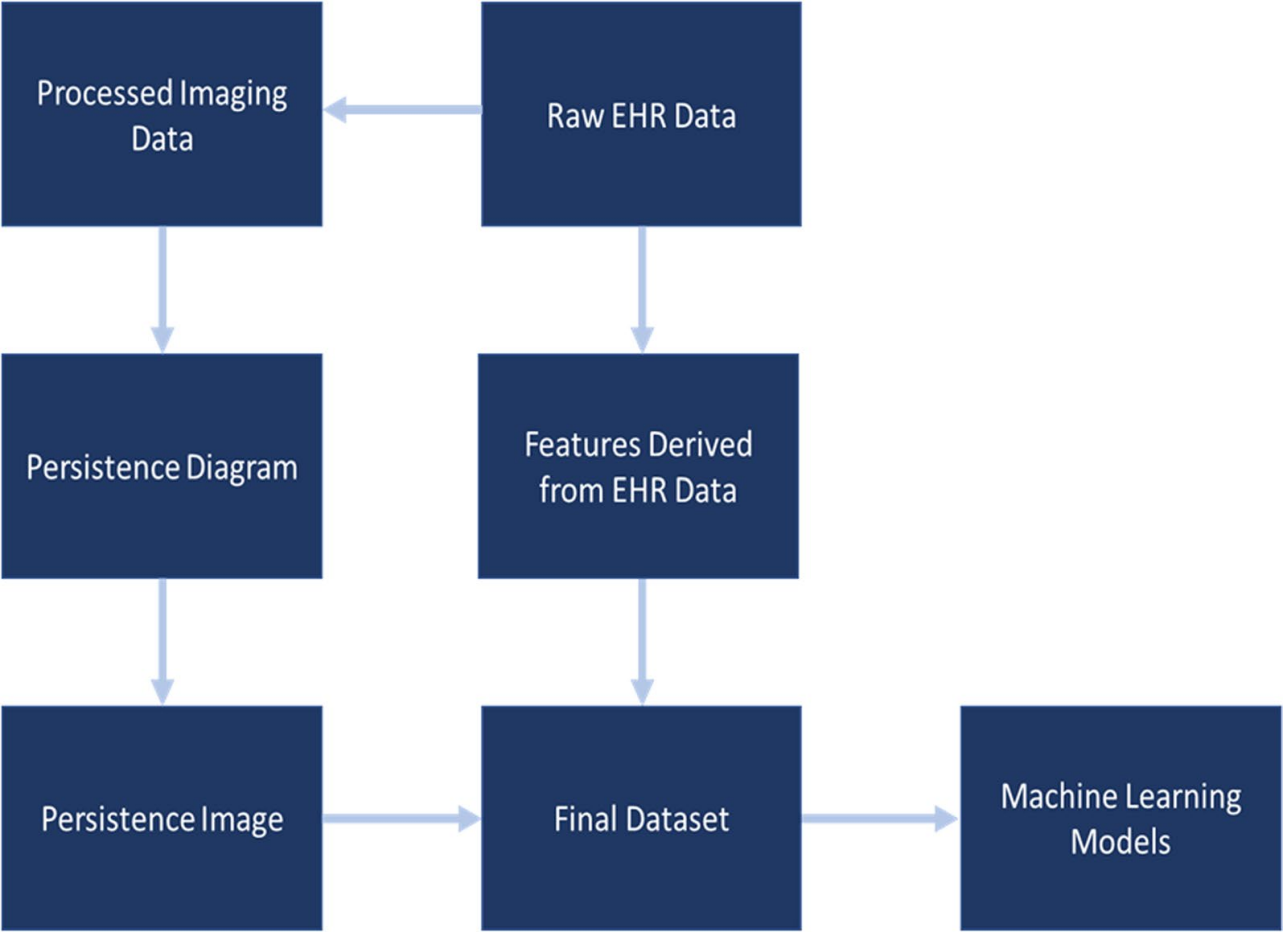
Workflow for integrating PH from imaging data and raw EHR data into machine learning models

However, imaging data features often need to integrate with other data sources or need to be combined within a multivariate model for further analysis. To do this, we need to transform the persistence diagram into a structure that will integrate well with statistical models or machine learning algorithms. Persistence images impose a weighting function to the points in a persistence diagram and then define probability distributions on those points. This gives a surface over the diagram, which becomes a feature vector for algorithms further down the analysis pipeline. In this way, persistent homological features derived from a filtration over a patient imaging dataset become features in a machine learning algorithm much like patient biometric measurements or demographic factors. Thus, we can think of PH and persistence images as a type of feature engineering like how features such as height and weight can be combined into a single metric of body mass index or how key words indicating a specific condition or medication can be flagged in an electronic health record (EHR). Figure 4 illustrates how this flow might work for raw EHR data.

## Traditional topological data analysis pipeline

It is assumed that the input consists of a finite set of points (pixel values) from medical images with a defined distance—or similarity—between them. This distance can either be an intrinsic metric determined by a pairwise distance matrix or induced by the metric on the point cloud (for example, the Euclidean metric when the data are embedded in R^d^). Typically, the definition of the metric is provided as an input or is dictated by the application. Different metrics can result in the extraction of varying features, which is crucial to define according to specific projects and clinical needs.

From the data, a “continuous” shape is constructed to draw attention to the underlying topology or geometry. This is frequently a simplicial complex or a family of nested simplicial complexes, known as a filter, which depicts the data's structure on several scales. Defining structures that can be successfully deployed in practice is the challenge at hand. The Vietoris–Rips algorithm tends to meet computational constraints of building hierarchies of simplicial complexes while preserving most features [18].

The structures constructed from the data are used to extract topological or geometric information. This may lead to either a complete reconstruction of the shape underlying the data—typically a triangulation—from which topological or geometric features can be easily extracted, or it may lead to rough summaries or approximations from which the extraction of pertinent information necessitates the use of particular techniques, like PH or Mapper. The problem at this stage is to (1) demonstrate the relevance of the topological/geometric information found, (2) include presentation and interpretation, and (3) show stability in the face of perturbations or the existence of noise in the input data. Understanding the statistical behavior of the inferred features is crucial for that goal as well.

New families of features and data descriptors are provided by the extracted topological and geometric information. They can be paired with other types of data for more in-depth analysis, or they can be utilized to explore the data through visualization. For instance, we can combine functional MRI data with clinical history notes, sociodemographic data, and biometric data on groups of patients to test the efficacy of a new traumatic brain injury drug. At this stage, it is crucial to demonstrate the added value and complementarity (in relation to other aspects) of the information provided utilizing TDA technologies.

## TDA in CT imaging

**Fig. 5:**
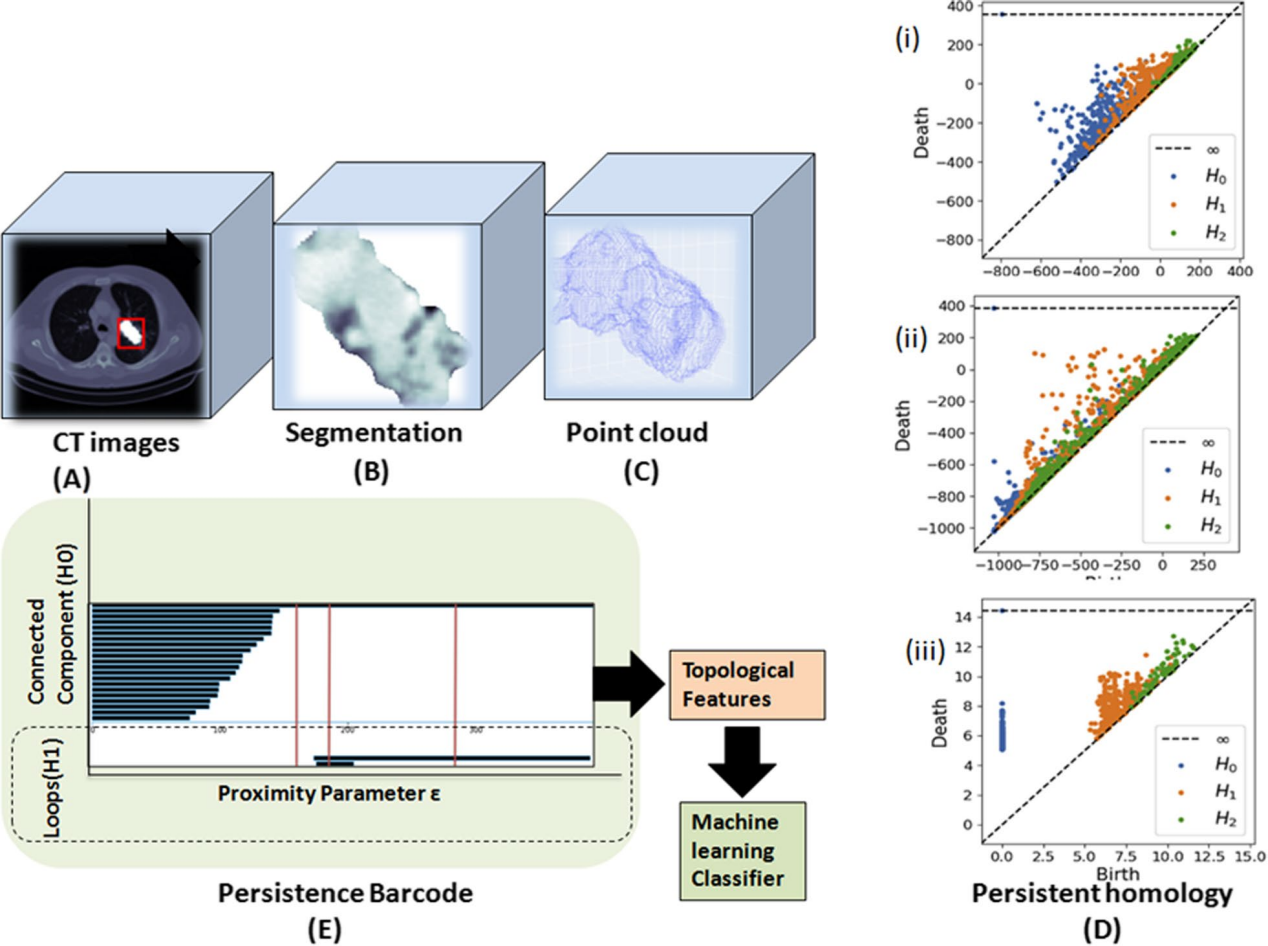
Numerous ways to compute PH from radiographic images. **a** An example 3D slice from a CT scan showing a lung tumor. The red box shows the lung tumor. The segmented tumor pixels are highlighted in white to distinguish them from their CT pixel values, which may be better seen in the following two images. **b** The same slice of the CT scan image only showing the tumor pixels that have been segmented. **c** A point cloud illustrating the tumor surface by stacking the tumor contours of all the 2D CT scan slices. **d** (i) Persistence diagrams derived from sublevel filtration of a 3D tumor image; image **b** showing a 2D slice. Three persistence diagrams are displayed. Each of the three dimensions of the topological hole under consideration has an unique diagram (*H*_0/0_-dim: connected components, *H*_1/1_-dim: cycles, and *H*_2/2_-dim: voids). (ii) The persistence diagrams, of which a 2D slice is shown in **b**, were generated by sublevel filtering the 3D tumor image with adjacent boundary box pixels. (iii) The lightly drawn persistence diagrams for the Vietoris–Rips filtering of the tumor surface-representing point cloud in **c**. **e** This is the persistent barcode extracted from the PH (H_0/0_-dim: connected components, *H*_1/1_-dim: cycles)

Current techniques for assessing texture patterns that result from local intensity change can only capture the spatial arrangement of the texture structures in 3D CT images. However, the main advantage of TDA is in offering a practical representation tool for comprehending and analyzing the spatial configuration of a 3D image texture component. For instance, in pulmonary nodules, the shape and connectivity of convex excursion sets can be expressed in terms of scalar quantities to capture the spatial arrangement of the texture of lung adenocarcinoma in great detail. The total amount of functionals needed to complete this task equals the excursion set's dimensionality plus one [19]. Regarding the geometric interpretation of the Minkowski functionals (MF) of the object under study in three dimensions (set of voxels in an image), the first functional corresponds to its volume, the second functional to its surface, the third functional to its mean integral curvature, and the fourth functional to the Euler–Poincaré number, which is a purely topological quantity [19]; the spatial configuration of the texture of a lung adenocarcinoma could be captured in great detail using this method. Boehm et al. [20] presented a TDA approach to express the spatial arrangement of textural feature maps in 3D images. This approach clarifies the geometric aspects of data from topology (Fig. 5) [19].

Another example of how TDA has been implemented on clinical CT imaging is assessment of survival rates of lung cancer patients, as shown by Somasundaram et al. [21], using persistent homology summary aspects of CT images. A cubical filtration based on Hounsfield units was created for each scan. The number of 0-dimensional topological characteristics was plotted against each Hounsfield unit to construct a feature curve and showed patients with lung cancer, with the 0-dimensional topological feature curve statistic indicating prognosis. While valuable, the use of TDA should be compared against other data modeling approaches to assess the superiority, or inferiority, of the technique.

In addition, Vandaele et al. [22] revealed how to predict the histology of lung tumors from thoracic radiography images using TDA and highlighted the advantages of TDA over cutting-edge quantitative imaging technologies for all the notable learning issues on lung tumor CT images. On thoracic radiographic images of lung cancers, this study investigated fundamental learning problems where PH outperforms the most recent radiomics-based learning techniques. An interesting finding was that the novel topological features captured complementary information well for “benign versus malignant” and “adenocarcinoma versus squamous cell carcinoma” tumor prediction but less consistently for “small cell versus non-small cell”. Radiomics is currently unable to characterize the overall data structure [22]. Furthermore, topological features appear to be superior to radiomics features in predicting tumor histology as determined by long-term radiology review, biopsy, surgical resection, progression, or response, even though radiomics features appear to be superior in predicting malignancy scores assigned by expert radiologists based on visual inspection [22].

In other work, Iqbal et al. [23] identified SARS-CoV-2 by computing their topological properties through CT images. To calculate the topological properties of SARS-CoV-2 features, PH from TDA was used to compute these topological features. The “SARS-CoV-2 CT scan dataset” [24], an open-source dataset with 2481 CT scans of healthy individuals and COVID-19 patients, served as the basis for the model's training and testing. The model achieved a benchmark F1 score of 99.42% overall, 99.416% in accuracy, 99.41% in precision, and 99.42% in recall.

## TDA in MRI

Topological properties can be extracted from grayscale MRI scans by first transforming scans into binary images through applying a threshold to each pixel value and then applying PH or persistent images.

**Fig. 6:**
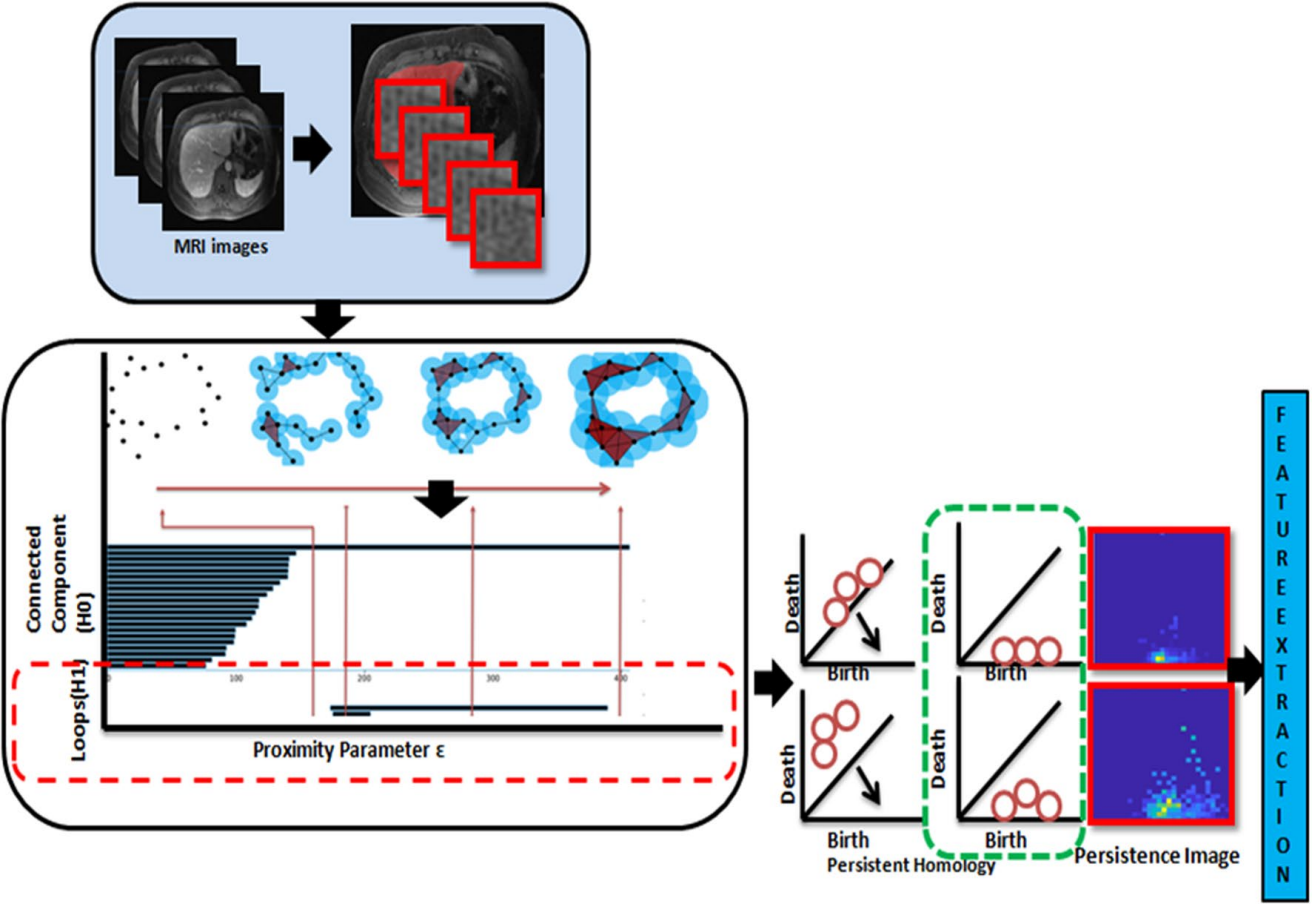
Workflow of algebraic topology-based machine learning with MRI imaging signal as input

Oyama et al. [9] investigated the accuracy for classifying hepatic cancers using PH to characterize T1-weighted MRI. By using algebraic topology-based machine learning, Singh et al. [25] extracted MRI features that predict the development of hepatic decompensation (Fig. 6) and also demonstrated the value of Betti numbers, which aid in the classification of liver diseases [26]. The topological features were employed as input for classification to predict who developed early hepatic decompensation within 1 year of their baseline MRI. When developed model was applied in the independent validation cohort, it remained predictive of early hepatic decompensation (AUC 0.84). In a different study, Turner et al. [27] developed the smooth Euler characteristic transform (SECT), a variant of the persistent homology transformer (PHT), to overcome the challenges of integration with conventional statistical models. SECT is a new statistic that enables the incorporation of shape information into conventional statistical models and was used to forecast disease-free survival in glioblastoma multiforme (GBM) based on tumor shape from post-contrast T1 axial MRI [28]. The output of PHT is a collection of persistence diagrams, whereas the output of SECT is a collection of smooth vectors [29]. Both create complex representations of the underlying topology that make it difficult to integrate with statistical models. Further statistical models, including the Bayesian linear mixed model (BLMM), have been employed in the identification of GBM [30–32]; these topological approaches performed gene expression, volumetric, and morphological summaries in predicting disease-free longevity when applied to GBM in MRI.

## TDA in ultrasound imaging

**Fig. 7:**
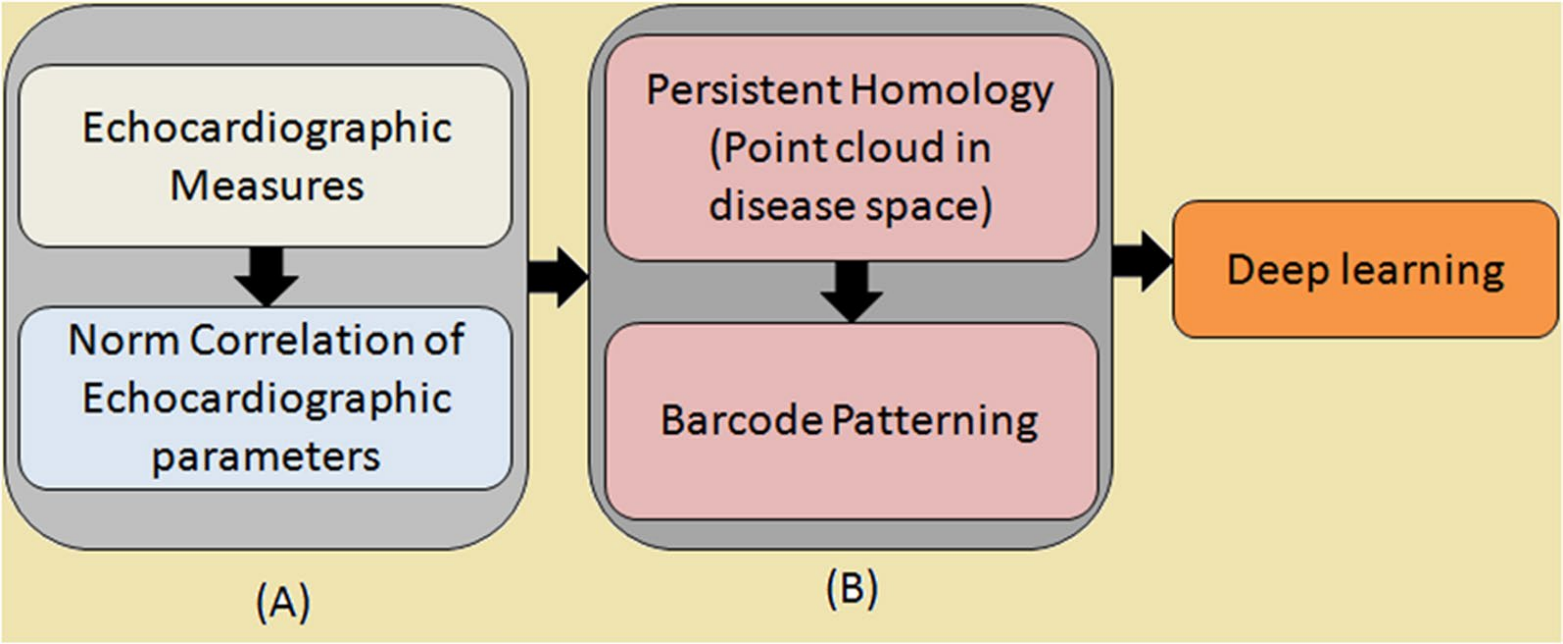
Workflow for echocardiographic features on TDA network. **a** Normalized bivariate correlation matrix of the different echocardiographic parameters of the dataset. **b** TDA combines the compressed representation with expressive visualization and understanding using a persistence diagram and barcode

Data is temporally and spatially normalized to an *n*-dimensional point cloud with simplicial complexes built upon the point cloud from which topological invariants are extracted. Birth and death features are transformed into a persistent image to develop feature vectors and features are stored in a visual representation that can be directly interpreted by physicians/scientists or serve as input for machine learning. Feature selection and classification of patients is performed using machine learning [33, 34]. TDA has been used to find new classification schemes that provided more information about the evolution of diseases. Casaclang-Verzosa et al. [35] characterized the natural history of aortic stenosis, which has two unique moderate stenosis phenotypic manifestations as it advances from mild to severe stenosis, in the first application of TDA in cardiovascular research (i.e., moderate aortic stenosis with normal vs. reduced ejection fraction) (Fig. 7). The same group reported findings in an abstract on TDA’s ability to differentiate a variety of heart illnesses with varying severity. On the basis of common electrocardiographic measures including left ventricular ejection fraction, mass, and so on, four patient subgroups with clearly different major adverse cardiac event (MACE) outcomes were automatically identified using unsupervised machine learning with TDA.

## Future applications of TDA

The widespread use of cross-sectional imaging in modern medicine underscores the need for advanced analytic techniques to augment detection and phenotypic characterization of diseases. As new technologies have emerged, the amount of data that needs to be analyzed has significantly expanded and become much more complex, driving researchers to develop approaches that enhance current data curation approaches. With the use of TDA, a relatively new analytical technique, researchers have made significant advancements in the understanding (pathophysiological features, etiology, prognosis) of several diseases, including cancer, asthma, and chronic lung disorders. TDA uses the data's “shape” to draw out important information and has the capacity to be combined with other techniques (i.e., PH) to transform data into visually meaningful representations, removing the “black-box” nature of conventional deep learning algorithms.

The field of TDA has many more algorithms than just PH and persistent images, and the ability to integrate TDA tools into data pipelines invites many possibilities for future directions. For instance, integrating brain imaging data with electronic health record text notes, genetic data, treatment history, biometric measurements, and sociodemographic factors can create richer models of patient outcomes for neurological or mental health disorders such that patients can be optimally matched to treatments [36]. Through persistent images, imaging data collected over the course of a disease (such as cancers) can be included within longitudinal models, such as generalized estimating equations, to understand the evolution of a disease like glioblastoma.

Many other TDA algorithms exist. Tools like Morse functions and the Conley index [37] can be used to cluster data by metrics of interest (part of the Mapper algorithm). Sheaves can be used to understand glucose flow on PET scans to better understand behavioral decisions related to health behaviors [38]. Tools from Hodge theory, such as the Hodge-Helmholtz decomposition, can be used to understand types of flow within biological systems, such as brains or tumors (or used to understand patient movements through the healthcare system) [39].

Simplicial complexes themselves provide another avenue for further investigation. Many tools from network science (applied to graphs) can be extended to higher-dimensional simplicial complexes [40, 41]. Geometric tools such as graph Laplacians and Forman–Ricci curvature already exist for simplicial complexes [42], but many tools have not been extended yet. Extension of network science tools allow for the extraction and summary of other features across filtrations besides homological features [43], and this avenue may be fruitful for image analytics, where features like degree or betweenness centrality might be relevant to underlying disease processes (such as the analysis of neural pathways or tumor angiogenesis). As these tools develop, they will provide a richer set of features to integrate with other healthcare data to understand disease etiologies and personalize treatment plans to optimize patient care.

## Data Availability

Not applicable.

## Abbreviations

AUC: Area under the curve
BLMM: Bayesian linear mixed model
CNNs: With convolutional neural networks
CT: Computed tomography
GBM: Glioblastoma multiforme
HER: Electronic health record
MACE: Major adverse cardiac event
MF: Minkowski functionals
MRI: Magnetic resonance imaging
PH: Persistent homology
PHT: Persistent homology transformer
SECT: Smooth Euler characteristic transform
TDA: Topological data analysis
